# Building Better Bridges: Outcomes of a Community-Partnered New School Transition Intervention for Students on the Autism Spectrum

**DOI:** 10.1007/s10803-024-06285-7

**Published:** 2024-06-12

**Authors:** Heather J. Nuske, Tristram Smith, Lynne Levato, Briana Bronstein, Nicole Sparapani, Consuelo Garcia, Fernanda Castellon, Hyon Soo Lee, Sarah F. Vejnoska, Samantha Hochheimer, Amber R. Fitzgerald, Jenny C. Chiappe, Amanda Dimachkie Nunnally, Jennica Li, Wendy Shih, Ashlee Brown, Michelle Cullen, Lisa M. Hund, Aubyn C. Stahmer, Suzannah Iadarola, David S. Mandell, Elizabeth McGhee Hassrick, Sheryl Kataoka, Connie Kasari

**Affiliations:** 1 Penn Center for Mental Health, Perelman School of Medicine, University of Pennsylvania, Philadelphia, PA USA; 2 Division of Developmental and Behavioral Pediatrics, University of Rochester Medical Center, Rochester, NY USA; 3 Center for Education, College of Health & Human Services, Widener University, Chester, PA USA; 4 Departments of Psychiatry and Behavioral Sciences, Psychology, and Education, UC Davis MIND Institute, University of California, Davis, Sacramento, CA USA; 5 Department of Psychiatry, Center for Autism Research & Treatment, UCLA Semel Institute, Los Angeles, University of California, Los Angeles, CA USA; 6 Teacher Education Division, Special Education, College of Education, California State University, Dominguez Hills, Carson, CA USA; 7 Professional Development and Research, Early Learning Services, Elwyn, Philadelphia, PA USA; 8 Special Education Department, Rochester City School District, Rochester, NY USA; 9 Health Resources and Services Administration, Division of Healthy Start and Perinatal Services, Maternal and Child Health Bureau, Rockville, MD USA; 10 A.J. Drexel Autism Institute, Drexel University, Philadelphia, PA USA

**Keywords:** School transitions, Parent coaching, Transition planning, Team coordination, Community-partnered, Social determinants of health

## Abstract

New school transitions can be challenging for students on the autism spectrum. No published, evidence-based interventions exist to support families and teachers of students transitioning to elementary and secondary school during this critical period. Using Community Partnered Participatory Research, we developed *Building Better Bridges (BBB)*, a caregiver coaching intervention that includes training on effective school communication, educational rights, advocacy, and child preparation strategies. We compared BBB (*n* = 83) to a module/resources-only comparison (*n* = 87) in a four-site randomized controlled trial in racially and ethnically diverse, under-resourced communities. In our intent-to-treat analysis, caregivers and teachers in BBB rated students’ transitions to the new classroom as more positive, relative to the comparison group. Results suggest this low-cost intervention can improve the transition process for families and students at high risk of poor transitions.

**Supplementary Information:**

The online version contains supplementary material available at 10.1007/s10803-024-06285-7.

For any student, transitioning from one stage of schooling to another, such as from preschool to elementary school or from elementary school to secondary school, can be challenging. For students on the autism spectrum, these challenges in new school transitions may be especially pronounced because of social communication differences, preferences for sameness and consistency, difficulties tolerating uncertainty (Boulter et al., 2014), and difficulty navigating complex social environments (American Psychiatric Association, 2013; Boulter et al., 2014; Cuccaro et al., 2003). Three recent systematic reviews found that students on the autism spectrum often experience increased anxiety, difficulties with self-regulation, and increased social pressure around school transitions (Fontil et al., 2020; Marsh et al., 2017; Nuske et al., 2019). Their caregivers (we use the term ‘caregivers’ broadly to refer to parents and other caregivers) often feel overwhelmed with complex placement decisions and worry about their children’s wellbeing (Fontil et al., 2020; Nuske et al., 2019). Teachers also struggle, feeling ill-equipped to provide appropriate support, often with inadequate resources (Fontil et al., 2020; Nuske et al., 2019).

The most useful transition strategies identified for students involved visiting the new school ahead of time, using visual schedules/calendars and social supports (peer buddies, designating a safe person/space), and supporting self-regulation or coping strategies (Marsh et al., 2017; Nuske et al., 2019). For parents, the most useful strategies included placement identification, included use of a transition binder that describes the practical steps throughout the school year, fostering communication between the home and both pre-transition (before the transition) and post-transition (after the transition) schools, linking with community organizations and parent support networks, and empowering parents to advocate for their child wellbeing (Fontil et al., 2020; Marsh et al., 2017; Nuske et al., 2019).

Fontil et al. (2020) highlighted that in particular, collaboration between teachers and families was often described as the most important facilitator of successful school beginnings. Marsh et al. (2017) emphasized that while parents and teachers were found to be highly involved in the transition process, transition supports were generic and rarely individualized to each student’s particular needs. In previous research, we found that teachers’ perceptions of successful student new school transition planning was directly associated with the size of their support network including connections between school and home (Dimachkie Nunnally et al., under review), such that teams with more perceived support had better planning.

Appropriate supports for teachers and their students on the autism spectrum can facilitate new school transitions. Although previous reviews have included sections on transitions supports, results here were observational and largely qualitative, focusing on the reported facilitators to successful new school transitions (Fontil et al., 2020; Marsh et al., 2017; Nuske et al., 2019). To our knowledge, only one intervention specifically designed to address the challenges posed during inter*-*school transitions of students on the autism spectrum has been evaluated to date—the Systemic Transition in Education Program for Autism Spectrum Disorder (STEP-ASD; Mandy et al., 2016). STEP-ASD is a manualized program for students on the autism spectrum in general education programs who are transitioning from into secondary school. The STEP-ASD manual contains a ‘transitions pack’ with information and resources for school staff, including how to familiarize the student with the new school and other ways to increase the predictability of the educational environment, chapters on specific core (e.g., social interaction difficulties) and associated (e.g. executive function difficulties) features of autism to increase teachers general knowledge about autism and provide practical, school-based support strategies, with associated resources provided in appendices (for more details see Mandy et al., 2016). A quasi-experimental study found that STEP-ASD reduced school-reported emotional and behavioral difficulties among public school students on the autism spectrum without a diagnosed intellectual disability (Mandy et al., 2016). To date, no such intervention has been evaluated for students on the autism spectrum who are transitioning to primary school or who have co-occurring intellectual disability, or for supporting caregivers and teachers in under-resourced communities or within predominantly minoritized populations (e.g., including people of color and families with limited financial resources). We use the term “minoritized” instead of minority to acknowledge that systemic inequities place individuals into a minority “at risk” status (Flanagin et al., 2021; Sotto-Santiago, 2019). The disparities that minoritized families of children on the autism spectrum experience in service availability and access (Angell et al., 2018; Mandell et al., 2009) point to the critical need for interventions to be developed specifically with the needs of these families in mind.

Indeed, some coaching interventions for caregivers of children on the autism spectrum have targeted caregiver knowledge and empowerment in their child’s special education program (e.g., Luelmo et al., 2021; Magaña et al., 2017), but none to our knowledge have focused specifically on school transitions. Involving caregivers is critically important, given that caregiver engagement in their child’s intervention is associated with greater consistency in use of intervention strategies across home and school (Crosnoe, 2015; McWilliam et al., 1999) and more effective strategies for problem solving (Newmann & Wehlage, 1995).

New intervention programs often are challenging to implement in schools; barriers include lack of leadership buy-in, limited resources, and inconsistent procedural fidelity (Iadarola et al., 2015; Langley et al., 2010). One way to address these barriers is to develop meaningful partnerships with community stakeholders, including those who will be responsible for adopting, implementing, and sustaining the program (Pellecchia et al., 2018). Community Partnered Participatory Research (CPPR) is a framework designed to increase meaningful collaboration and shared decision-making between communities and academic institutions (Jones, 2018) that has successfully facilitated implementation of various health-related interventions in communities (Hankerson et al., 2018; Wells et al., 2013). Authentic partnership also supports health equity by ensuring that the research relationship includes members of minoritized communities and that their perspectives are integrated into all facets of the work (Wells & Jones, 2009).

## Developing and Refining the *Building Better Bridges* Intervention

To address the gaps in the literature, we developed a transition-focused intervention, called Building Better Bridges (BBB), that emphasized caregiver engagement. We began by having the CPPR developers (L. Jones) train the study investigators. We then formed community partnerships at study sites (Los Angeles, Davis, Philadelphia, and Rochester). Our partners provided input at each step of the intervention development and study. Partnership teams included family members who had children or youth on the autism spectrum, autistic youth themselves, service providers involved with school-based transition processes in preschools and school districts serving minoritized families, school district administrators, early intervention system providers, teachers, specialized service providers, and other agency representatives. At some sites, partners also included community organizations with a broader focus on child, family, and community-wide initiatives and those providing outreach to specific racial and ethnic groups. Partnership meetings occurred monthly throughout intervention development and recruitment, with some sites engaging in shared community-academic leadership facilitating each meeting and others had academic partners leading meetings. Locations of meetings also varied by site, depending on the needs of partners, either alternating between the university campus and community locations, or meeting exclusively in the community. Partners engaged in discussion about the study processes, co-developed intervention materials and reviewed materials in development, shared recruitment ideas, and discussed ways to troubleshoot barriers. All study design, protocols, recruitment, and implementation for this study was collaboratively produced by the community research partnership, including this manuscript.

Second, as described above, we conducted a systematic literature review on studies of school transitions in students on the autism spectrum (Nuske et al., 2018). Third, we conducted focus groups and interviews with teachers and caregivers of students on the autism spectrum to understand their needs in transitioning students on the autism spectrum across school systems. Caregivers identified supportive strategies, including school tours, transition workshops, meetings with staff about the upcoming transitions, and transition skills for the youth as important to supporting new school transitions (Smith et al., 2021). Both caregivers and teachers identified the importance of building and maintaining school teams that include caregivers, pre-transition teachers, post-transitions teachers and other providers for successful transitions (Smith et al., 2021), which is consistent with prior systematic reviews (Fontil et al., 2020; Marsh et al., 2017; Nuske et al., 2019).

Integrating these findings helped us develop a first draft of BBB, which we then pilot tested. In this pilot, we included a “transition facilitator,” a school staff member at the pre-transition school, who would receive BBB coaching and resources to guide the student and their family through the school transition. During the pilot, however, we found that school staff were often reluctant to take on the role, feeling that it was not within the scope of their employment or capacity to support students once they transitioned out of their school. We, therefore, re-conceptualized the intervention as a caregiver-implemented intervention in which the student’s caregiver was the “transition facilitator,” who would receive coaching from the research team, since the caregiver was the constant person on the student’s school team who could support the student from one school context to the next. This reconceptualization follows recommendations of systematic reviews that emphasized caregivers’ role in facilitating successful school beginnings and the importance of collaborative practices (Fontil et al., 2020; Marsh et al., 2017; Nuske et al., 2019).

The revised BBB intervention included modules that supported caregivers (“caregiver modules”) in advocating for their student by gathering key information from pre-transition teachers about their student to pass on to post-transition teachers. Caregivers also learned how to communicate effectively with school teams, gained knowledge about educational policy and their rights related to school transitions, and learned strategies for assisting their student in preparing for the transition in partnership with a coach to support their use of the BBB materials. See Methods, ‘*Building Better Bridges* Intervention’ section for more details.

## Current Study

The current study tested BBB compared to a non-coaching comparison intervention including access to printed material of caregiver modules in a resource binder, in a randomized controlled trial (RCT). The RCT examined the impact of BBB on school transition-related outcomes for students on the autism spectrum, their caregivers, and teachers. As described elsewhere (Wells et al., 2018), our use of CPPR helps to address key implementation factors described in each of the five key areas of the RE-AIM framework (*Reach, Efficacy, Adoption, Implementation, and Maintenance*; Glasgow et al., 1999). In this study, *Reach* of minoritized families of students on the autism spectrum and in under-resourced school districts was facilitated by our community partnered approach. For example, BBB emphasizes coaching caregivers in their preferred language using culturally salient examples and recruiting minoritized and rural students and families who often do not have access to needed autism-related services. *Efficacy* of BBB was the main focus in the current study. We attempted to maximize this in the development phase of the intervention by incorporating input from educators, students on the autism spectrum, and family members on best practices for school transitions. Community partners influenced the intervention to be *Adoption* ready, by providing insights into structural barriers within schools and highlighting the importance of simplifying both intervention steps and resources. CPPR and the pilot study also guided our *Implementation* approach, with partners helping to refine our caregiver coaching implementation strategy, and relatedly, to an overall understanding of the need for some flexibility in accommodating families’ overcoming social stressors and logistical challenges. Finally, our partnership with school teams and leaders helped us design BBB for *Maintenance* in under-resourced, public school systems, with freely accessible, electronic resources and materials available in multiple languages, although data collection on *Maintenance* was outside the scope of the current study.

We hypothesized that, compared with students in the comparison group (caregiver module and resource binder only), students in the intervention group (caregiver module and resource binder + caregiver coaching) would benefit from more successful transition planning and have more successful transitions, and caregivers would experience more self-efficacy and less worry in managing the transition from one school setting to the next. We also hypothesized that caregivers and teachers in BBB would find the intervention more usable and acceptable compared with those in a comparison group, who were provided materials (i.e., the caregiver module and resource binder) without coaching support.

## Methods

### Design

We tested the BBB intervention versus comparison in an RCT over two academic school years. Teachers were randomized to either BBB intervention or a caregiver module and resource only comparison group that did not include individualized coaching on the caregiver modules (see Intervention Components and Groups section). Initial randomization was stratified based on the number of consented students within each teacher’s classroom (one versus two or three consented students). Caregivers were given the same random assignment as their teacher in their dyad. We used this randomization approach to randomize teachers rather than parents (as teachers were also involved in the intervention, as explained below) to ensure no contamination across the study arms, as teachers could have up to three parents involved in the study (e.g., to avoid parents discussing coaching with other parents who were not receiving coaching). The study included three main data collection time points: (1) Baseline: at study enrollment (pre-intervention); (2) Pre-transition: the last 6 weeks of pre-transition school year; and (3) Post-transition (post-intervention): after the first 6 weeks of the new school year.

### Setting

This study was conducted through the Autism Intervention Research network on Behavioral Health (AIR-B). Four sites participated: Los Angeles, California; Davis, California; Philadelphia, Pennsylvania; and Rochester, New York. Each site conducted the study in partnering with rural or Title 1 public schools. These included many urban schools, all with large proportions of minoritized families, including families of color and families with limited resources.

### Recruitment

**Fig. 1:**
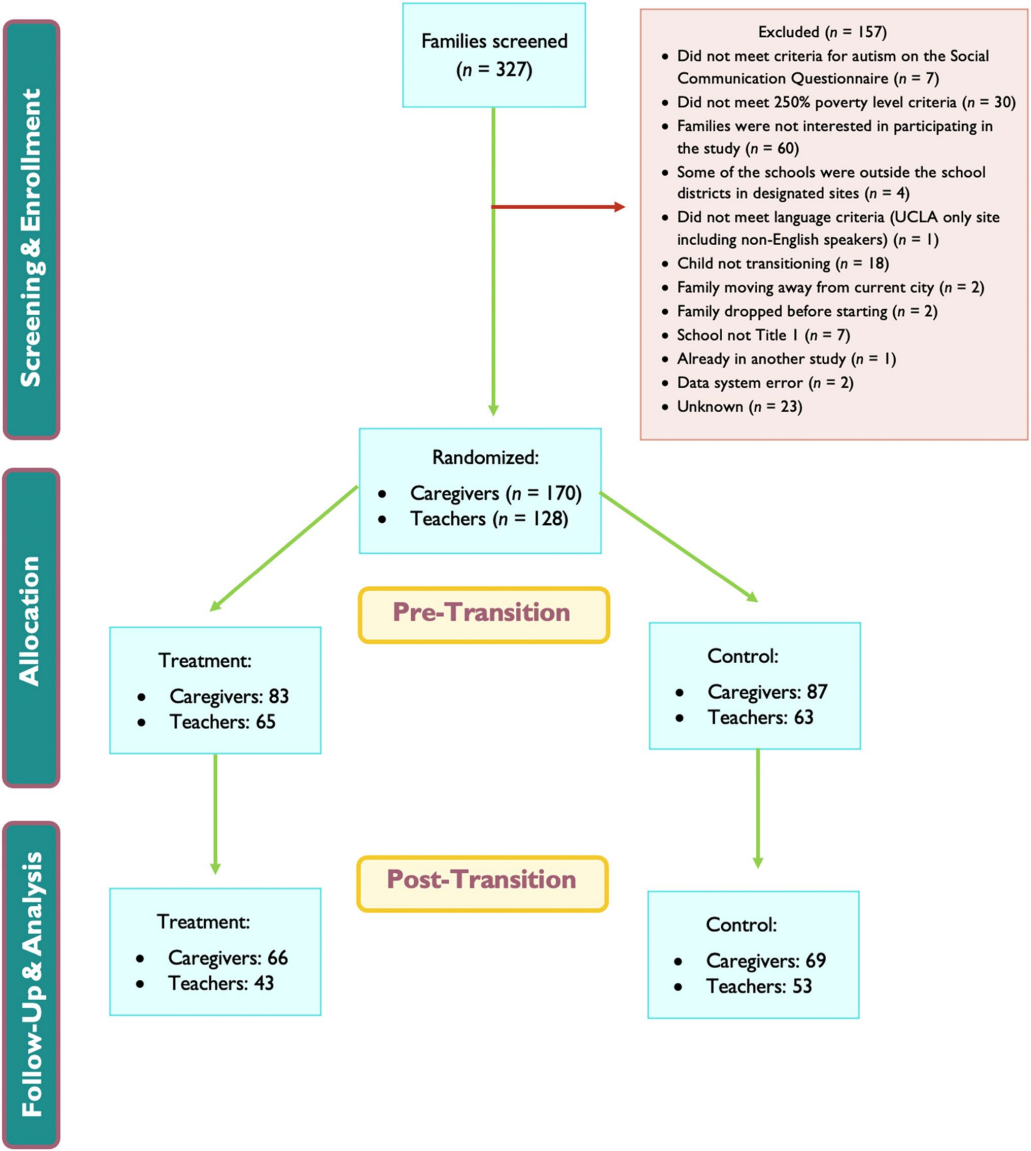
Participant screening and recruitment

With the help of our community partners, and after seeking school administrator permission, we recruited caregiver-teacher dyads through meetings with principals, teachers, and/or school counselors, presenting details about the research study at local outreach events such as service fairs, back-to-school nights, caregiver information sessions and parent-teacher nights/conferences. We used a dual recruitment strategy whereby either teachers or caregivers were the first member of a dyad, and then we asked them to reach out to the second member of the dyad to share our study invitation. School personnel shared recruitment materials with potentially qualifying students and families. Interested families who consented to be contacted by the research team completed a study screening process. See Fig. 1 for CONSORT diagram on screening and inclusion details.

### Participants

#### Caregivers and Students on the Autism Spectrum

A total of 170 caregivers of students on the autism spectrum participated in the study over the course of two school years. Inclusion criteria were as follows: (1) the student had to be transitioning into elementary or secondary school (middle/high school) the upcoming academic year; (2) both a caregiver and at least one member of the student’s pre-transition educational team were required to participate; (3) the student had a medical diagnosis or educational classification of autism; (4) the student had to meet the cutoff score of 12 for children under 8 years of age or 14 for children older than 8 years (Corsello et al., 2007) on the caregiver-completed Lifetime version of the Social Communication Questionnaire (SCQ; Rutter et al., 2003); and (5) families had to qualify as “under-resourced,” which we operationalized as attending or transitioning to a Title I school (federally funded educational program for school districts with large concentrations of families with limited financial resources) or rural school and having a family income less than 250% of the US federal poverty line for 75% of the sample (with 25% of the sample under 400% US federal poverty line). Due to the research team’s capacity, families had to speak English or Spanish (or Korean at the Los Angeles site). Sixteen families received the intervention in Spanish and one in Korean in the Los Angeles area. For the Spanish participants, all study and intervention materials including the measures and the resource binder content were translated by bilingual research staff, and Spanish-speaking coaches were assigned to caregivers that only spoke Spanish. For the Korean participant, a bilingual research staff member scheduled extended meetings with the family to review the consent procedure and survey items in Korean. The family agreed to receive the materials in English. All intervention procedures remained the same.

89% of caregivers identified as female, with an average age of 38.7 years (*SD* = 9.8). A plurality (44.1%) of the caregivers identified as White, 20.6% identified as Black/African American, 4.1% identified as Asian American/Pacific Islander, 1.8% identified as Indian/Alaska Native, 1.2% identified as Native Hawaiian/Pacific Islander American, 4.1% identified as Multiple Races/Black, 17.7% identified as Other (e.g., Guatemalan, Puerto Rican, Filipino), and 6.5% preferred not to answer. A little more than half of the caregivers (52.3%) identified as Hispanic/Latinx.

Students’ average age was 7.8 years (*SD* = 3.8) and average SCQ score was 21 (*SD* = 5.6). Students’ IQ ranged from 38 to 120, with an average of 73 (*SD* = 20.9). If students had a recent IQ score on file from a recognized standardized IQ battery administered by their educational or medical team (e.g., Wechsler, Differential Ability Scales, Stanford Binet), from the previous year for preschool students or from the previous 0–3 years for elementary and middle school students, this score was used; otherwise the research team administered the Stanford Binet Abbreviated Battery IQ scales (Roid, 2003). In keeping with the study aims, 75% of families earned less than USD $50,000 per year, 17% earned USD $50,000–$79,999, and 8% earned USD $80,000 or more. 54% of students were transitioning to elementary school, 28% to middle school, and 18% to high school.

#### Teachers

A total of 128 pre-transition teachers and 96 post-transition teachers participated. Demographics for teachers are presented in Table 1.

#### Baseline Group Matching

There were no differences between the intervention and comparison groups on any caregiver, student, or teacher demographic variables (*p* range = 0.11−091).

**Table 1:** Teachers’ demographic characteristics

	Pre-transition	Post-transition
*N*	128	96
Average age	41.3 years (*SD* = 10.8)	40.1 years (*SD* = 10.3)
Gender (female)	87.5%	82.3%
Race/Ethnicity		
White	71.9%	72.9%
Black/African American	7.8%	3.1%
Asian American/Pacific Islander	3.1%	2.1%
Native Hawaiian/Pacific Islander American	0%	1%
Multiple Races	2.3%	6.3%
Other	3.9%	4.2%
Prefer not to answer	10.9%	10.4%
Hispanic/Latinx	53.9%	37.5%
Teacher statistics		
Special education teacher	84.4%	83.3%
General education teacher	8.6%	15.6%
Other specialist (special education coordinators, speech-language pathologists, social workers, resource specialists, school psychologists, and behavioral therapists)	7%	1%
Average years in current role	8.9 (*SD* = 7.7)	9.8 (*SD* = 8.7)
Previous experience working with students with autism	93.8%	96.9%

**Table 2:** BBB coaching modules

Coaching module topic	Description
Developing the Student Snapshot	Collaborating with the pre-transition team to document the key information that post-transition teachers will need to know about their child (see ‘Key student information page: Student Snapshot’ section); conducted with the child’s teacher if possible
Understanding the Educational Team	Identifying members of the students’ school team, the role of each team member and services provided and potential members of the post-transition team
Knowing Your Rights	Identifying important transition related special education laws and policies, including (US-specific) the Individuals with Disabilities Education Act (IDEA), Free Appropriate Public Education (FAPE), the Individualized Transition Program (IEP), and the Individualized Transition Plan (ITP).
Gathering New School Information	Identifying the new school and setting a plan to get additional information including visiting the school if possible
Preparing Your Child Over the Summer	Reviewing options for activities/visual supports to prepare child over the summer based on child’s needs (e.g., social narratives about the new school or transition, a first day equipment list, self-regulation exercises like a calm-down checklist and a My New Locker activity)
Sharing the Student Snapshot	Tips for sharing the Student Snapshot with the new school team

### Intervention Components and Groups

#### Intervention Group: Building Better Bridges (BBB)

The intervention included (1) a BBB Resource Binder including content from each of the six caregiver modules, among other resources, and (2) Individualized Coaching on the caregiver modules.

##### BBB Resource Binder

The BBB Resource Binder consisted of materials created and adapted for the BBB intervention, namely, the six caregiver modules and associated resources. Module topics are each described in Table 2. Briefly, the caregiver modules addressed topics like establishing effective communication with school teams, understanding caregiver rights in special education and educational policy related to school transition, caregiver advocacy, and activities to complete over the summer to prepare students for transition to their new schools. Complementary resources within each module included checklists (e.g., for gathering information about the new school), tips for preparing the child over the summer, as well as general resources related to transition (e.g., a transition planning checklist). State and city specific resources and supports, such as local caregiver support groups and autism services within the geographic location were also available in the binder. All caregivers and teachers, no matter the intervention group, received a paper copy of the binder and an online version, administered via Livebinder, an online binder website. The binders are available for free download here: https://www.airbnetwork.org/downloads/#buildingbetterbridges.

**Table 3:** Transition evaluation questionnaire: pre-transition and post-transition versions

Pre-transition version items	Post-transition version items
1. How effective has communication been about the transition process between you and the child’s *current* school team during this school year?	1. How effective has communication been about the transition process between you and the child’s *pre-transition* school team during this school year?
2. How effective has communication been about the transition process between you and the child’s *future* school team during this school year?	2. How effective has communication been about the transition process between you and the child’s *new* school team during this school year?
3. Based on current transition planning, how successful do you think the child’s transition will be to his/her new school?	3. How successful do you think the child’s transition has been to his/her new school?
4. How much transition support planning has been provided to you by the child’s *current* school team during this school year?	4. How much transition support planning has been provided to you by the child’s *pre-transition* school team during this school year?
5. How much transition support planning has been provided to you by the child’s *future* school team during this school year?	5. How much transition support planning has been provided to you by the child’s *new* school team during this school year?
6. Do you feel that the child’s *future* school team will have the necessary information that they need from the current school team to begin working with the child in the fall?	6. Do you feel that the child’s *new* school team has the necessary information that they need from the pre-transition school team when they began working with the child in the fall?
7. How satisfied are you with the way the child’s current school team is preparing them for their future school?	7. How satisfied are you with the way the child’s pre-transition school team prepared them for their new school?

##### Student Snapshot

The Student Snapshot was included in the BBB Resource Binder, a two-page fillable document that consist of key information for each teacher to review about the student transitioning into their classroom to facilitate transition. This included student strengths/interests, reinforcing items, and how they communicate best. It also had a place for the students’ triggers, behaviors, and best strategies for addressing them. The caregiver’s dreams and aspirations for their child were included, as well as health and safety concerns, and any relevant teaching tips.

##### Individualized Coaching

**Fig. 2:**
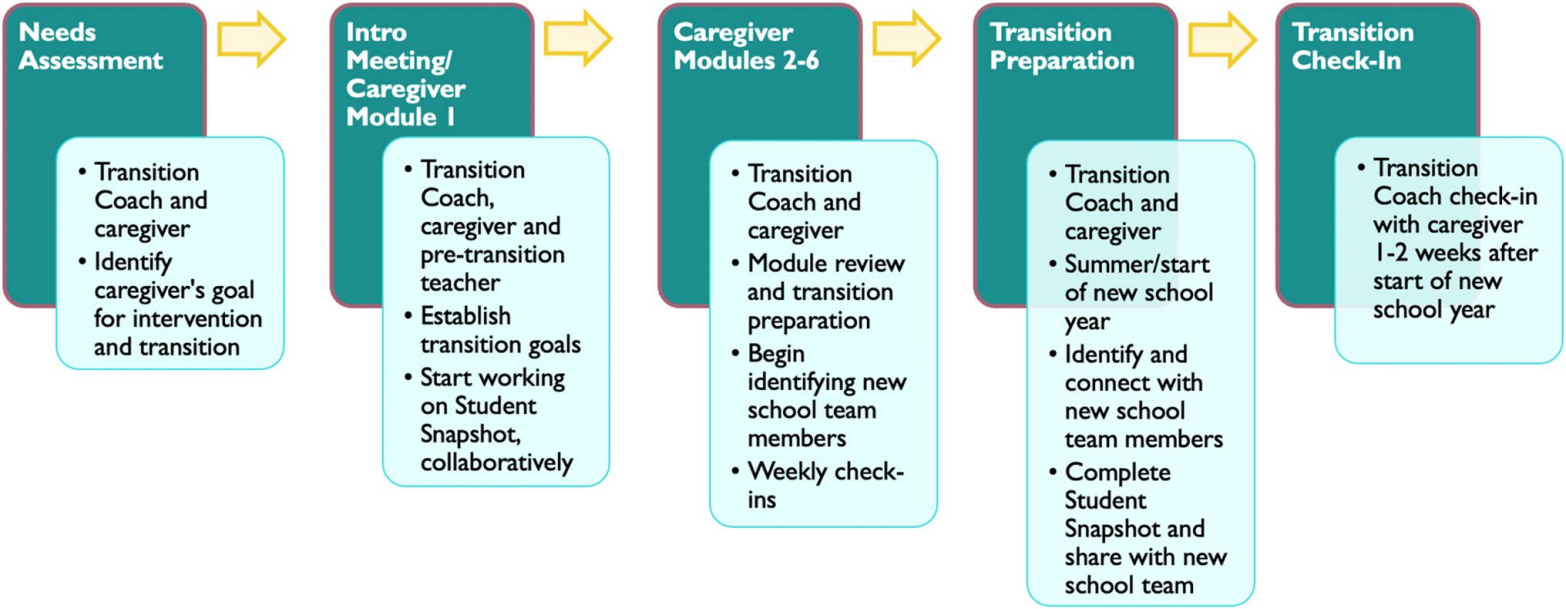
Transition Evaluation Questionnaire (TEQ) results showing higher overall transition preparation/success at Pre-Transition and Post-Transition in the intervention vs. comparison group as reported by caregivers (**A**) and higher overall transition preparation at Pre-Transition as reported by teachers (**B**). Note that the lines between Pre-Transition and Post-Transition teachers are broken as these represent different teacher groups (from pre-transition and post-transition schools, respectively)

In addition to the BBB Resource Binder, the intervention group received individualized coaching on the six caregiver modules. The coaching was delivered by a Transition Coach (a member of the research team with a Master’s degree or Ph.D. and autism experience) matched on the Transition Coach’s availability and the caregiver’s preferred language (e.g., English, Spanish, or Korean). To ensure consistency across coaches, coaches met weekly to discuss issues related to coaching, supervised by a licensed clinical psychologist on the team. See Intervention Procedures section for more details on coaching delivery and Fig. 2 for an overview of the intervention delivery.

Additionally, intervention group participants were invited to enroll in the *ParentSquare* application, a web-based team communication platform commonly used in schools. This was offered to participants as an optional way to securely communicate and share files, and to facilitate communication between the pre-transition teacher and the parents during the transition preparation stage, as well as between the pre-transition teacher, post-transition teacher and the parent at the transition stage. Each family randomized in the intervention group had their own individual *ParentSquare*. Transition Coaches used *ParentSquare* to communicate with the school team and caregivers by uploading the caregiver module documents, creating reminders, and posting weekly to bi-weekly check-in notes. All participants in the intervention group were given access to *ParentSquare* through the end of the calendar year of enrollment. For details on intervention group procedures, see Procedures section.

#### Comparison Group: BBB Resource Binder Only

All participants (caregivers and teachers) assigned to the comparison group received the BBB Resource Binder, including the six caregiver modules and local resources. They were encouraged to use the resources to facilitate students’ transition, but were not given any individualized coaching or access to *ParentSquare*. For details on comparison group procedures, see Procedures section.

### Measures

#### Primary Outcome: Transition Evaluation Questionnaire

We developed the Transition Evaluation Questionnaire (TEQ) questionnaire for this project to measure teacher and caregiver perceptions regarding school transition planning and success, and have previously used it in an observation study of transition networks (McGhee Hassrick et al., 2021) and a study on baseline measurement of teacher’s perceptions of upcoming school transitions (Dimachkie Nunnally et al., under review). We collected data on how pre-transition teachers, post-transition teachers, and caregivers of students on the autism spectrum prepared for transitions and used it to inform the questionnaire. The resulting questionnaire included 7 items, rated using 6-point Likert scales. See Table 3 for the full list of items (TEQ-Teacher version provided; the TEQ-Parent version used “your child” instead of “the child” across items, all other wording was identical). The total score was computed by calculating the (sum ÷ total possible score) × 100 (0–100 range), with higher scores representing higher transition success. Internal consistency on the TEQ total scores was high across caregiver and teacher respondents across the pre- and post-transition versions (M *α* = 0.83, range *α* = 0.71–0.89).

#### Secondary Outcome: Parent Self-Efficacy in Managing the Transition to School Scale

Caregivers completed the Parent Self-efficacy in Managing the Transition to School Scale (PSMTSS; Giallo et al., 2008), as a measure of self-efficacy and worry about handling their student’s school transition. Caregivers indicated how strongly they agreed or disagreed with nine statements about their child’s school transition process (e.g., “I have a clear understanding of what my child might experience as they move to their new school”; “I worry about my child’s adjustment in a new environment”) on a 6-point Likert scale ranging from 1= “Strongly disagree” to 6= “Strongly Agree” (Giallo et al., 2008). Five items formed the Efficacy subscale and four items formed the Worry subscale. Items were scored such that lower scores indicated less efficacy and worry, and higher scores indicated higher efficacy and worry. The two subscales, Efficacy and Worry, have been found to have good internal consistency (0.74 and 0.76, respectively; Giallo et al., 2008).

#### Implementation Outcomes

We included three implementation outcomes: coaching fidelity, intervention usability, and intervention acceptability. Each is described in detail below.

##### Coaching Fidelity

Coaching fidelity was assessed via a self-report form completed by the Transition Coach after each coaching session. Each item on the fidelity form was scored using a score of 0 (not completed/ implemented) 1 (partially completed/ implemented), 2 (fully completed/ implemented), or N/A (not applicable). Items included (1) reviewing objectives covered in the previous session, (2) checking in to see if any new problems or concerns were presented since last session, (3) checking in with caregiver about tasks completed since last session and if necessary coach assessed the level of support caregiver needed to complete previous tasks, (4) if necessary, assessing the level of support caregiver needed to complete previous tasks, (5) introducing topic of session, (6) providing an opportunity for the caregiver to share his/her feelings about the topic, (7) providing an opportunity for the caregiver to ask additional information or share concerns about the topic, (8) reviewing objectives for coaching topic, (9) following coaching script (with adaptations based on family need), (10) collaboratively determined next step tasks to be completed, (11) discussing strategies for completing the next steps and (12) determining support needed for caregiver to complete next steps and made a plan to put those in place (follow-up call, mailing materials or handouts). Coaches scored each item and calculated a total number of steps followed to calculate a percent followed per session. Inter-rater reliability was measured for 20% of sessions of each coaching case (randomly selected) coded by an additional observer from audio recordings of the session to measure fidelity.

##### Intervention Usability

Following participation in the study, caregivers and teachers completed scales on intervention usability to determine which, if any, intervention tools they used, which intervention meetings they attended (introduction meeting and transition planning meetings organized by the school) and how useful they found each to be, and for participants in the intervention group, how useful they found the coaching on the caregiver modules to be. Participants rated items about intervention tools, meetings and coaching on the caregiver modules on a scale where 1 = Not useful, 2 = Somewhat useful and 3 = Very useful, such that higher scores indicate higher levels of usefulness. Participants could also indicate if they did not use a particular intervention component by choosing N/A.

##### Intervention Acceptability

Following participation in the study, caregivers and teachers were also asked to rate their perceptions of the acceptability of the intervention. The measure used was originally created as part of a wider measure of implementation climate and was used to measure implementation climate of a school-based social communication intervention from the AIRB-3 network (Shih et al., 2019). An adapted version of the original measure was used in this study to focus on acceptability, and adjusting the language to pertain to the current intervention study. There are 10-items in total. Example items are: (1) I feel that the intervention was an appropriate intervention for this child; (2) I feel that the intervention improved this child’s transition; (3) I feel that the intervention was easy to implement; (4) I would recommend this intervention to other parents. See Supplementary Material for all items. Items were scored on a 5-point Likert scale: 1 = Strongly disagree, 2 = Disagree, 3 = Don’t Know/Neutral, 4 = Agree, 5 = Strongly Agree), such that where higher scores indicate higher acceptance of the intervention. The acceptability measure had high internal consistency reliability across parent and teacher versions (*α* = 0.95-0.97).

### Procedures

#### Intervention Procedures

##### Intervention Group

After consenting to the study, Transition Coaches first conducted a needs assessment with the caregiver for participants randomized to the intervention group to identify up to five goals for the intervention and transition. During this meeting, the Transition Coach assessed the caregiver’s feelings and concerns about the upcoming new school transition, the level of information the caregiver had received about the transition, how best the caregiver learns, and anything else the caregiver wanted to share regarding the upcoming transition. This information helped guide coaching on the caregiver modules through the rest of the intervention. For example, the Transition Coach would use information about the new school or teacher had or been identified yet to determine how to approach the caregiver module focused on gathering information about the new school and when would be most appropriate time to deliver this caregiver module. Participants and members of the student’s school team (including parents, teachers, and any other key members of the team) were added to *ParentSquare* (described above).

Following the needs assessment, the Transition Coach and caregiver met approximately monthly to complete coaching on the caregiver modules. The Transition Coach delivered the caregiver modules through an interactive PowerPoint presentation either in person or via phone or web conferencing software (Zoom). In-person sessions took place at the caregiver’s home, school, or in the community (e.g., local coffee shop/restaurant, local library, etc.) per the caregiver’s preference. Check-ins occurred approximately weekly to twice a month in between sessions according to the caregiver’s preferred method of communication (e.g., telephone, text, email, or *ParentSquare)*.

The caregiver module on developing the Student Snapshot ideally included the Transition Coach, caregiver, and pre-transition teacher or school personnel. The Transition Coach used this opportunity to encourage both caregivers and teachers to collaborate to provide input about the student and what might contribute to a successful transition. After this meeting, the remaining caregiver modules were completed by the Transition Coach and caregiver in an order that most suited the caregiver based on the needs assessment. Once the new school was identified, new school team members were recruited to participate and invited to join the team’s *ParentSquare.* Transition Coaches encouraged caregivers to share Student Snapshot the student snapshot with the new team as soon as possible. Finally, the Transition Coach checked-in with caregivers 1–2 weeks after the start of the new school year to ask how the transition went and to provide any final support to facilitate the transition. See Fig. 2 for an overview of the intervention delivery.

##### Comparison Group

After consenting to the study, participants randomized in the comparison group received a hard copy of the BBB Resource Binder and online access on Livebinder.

#### Data Collection Procedures

At baseline, measures were collected either in person during the meeting in which informed consent was obtained from each participant, or via an online survey. Caregivers also completed the demographics form, TEQ, and PSMTSS at this meeting. Teachers completed the demographics form and TEQ. At pre-transition the TEQ and PSMTSS were collected, and at post-transition the TEQ, PSMTSS, Intervention Usability and Intervention Acceptability forms were collected from caregivers and teachers. Pre- and post-transition measures were collected via an online survey. Families could request hard copies of the questionnaires be mailed to them with a pre-stamped return envelope if they preferred. At each time point, families received a $25 gift card once their measures were completed. Data from all sites were entered into an online data storage system built by the Semel Institute Biostatics Core (SIStat). The study was approved through each partnering site’s university and their local school district.

### Data Analysis

Data were checked for skewness and kurtosis and deemed normal so parametric analyses were conducted. For the primary and secondary outcome analyses, we conducted linear mixed models, with intervention group (intervention, comparison), time (baseline [pre-intervention], post-transition [post-intervention]), intervention group × time, and school level (elementary, middle, high) as fixed effects and site as a random effect, to examine the intervention effect of the BBB intervention on transition success as measured by caregivers and teachers (TEQ total scores), and caregiver’s self-efficacy and worry in managing the transition to scale (PSMTSS sub-scale scores). Bonferroni corrections were applied to correct for multiple testing. We also report descriptive statistics on the coaching fidelity, intervention usability and intervention acceptability forms, and exploratory analyses (in Supplementary Material) with Pearson correlations between binder component usefulness ratings and outcomes.

## Results

### Primary Outcome: Overall Transition Success

**Fig. 3:**
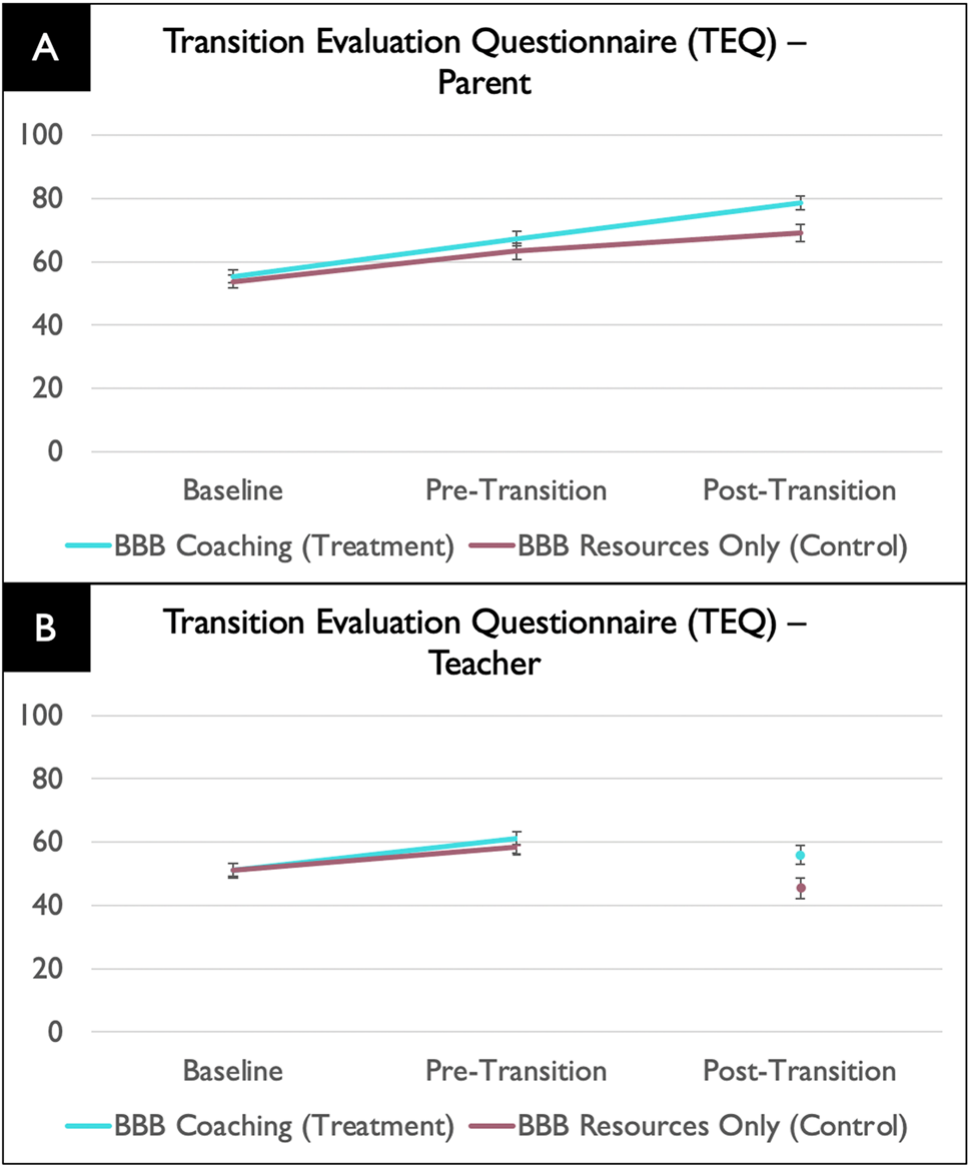
Transition Evaluation Questionnaire (TEQ) results showing higher overall transition preparation/success at Pre-Transition and Post-Transition in the intervention vs. comparison group as reported by caregivers (**A**) and higher overall transition preparation at Pre-Transition as reported by teachers (**B**). Note that the lines between Pre-Transition and Post-Transition teachers are broken as these represent different teacher groups (from pre-transition and post-transition schools, respectively)

For overall transition success (TEQ total score), there was a intervention group × time interaction effect on TEQ total, marginally for caregivers (*F*(1, 147) = 3.80, *p* = .053), and significantly for teachers (*F*(1, 195) = 4.24, *p* = .04). Pairwise (post hoc) comparisons showed that for both caregivers and teachers, there were no significant group differences in TEQ score at baseline (*p* = .64 and *p* = .91, respectively); however, there was a significant post-intervention (post-transition) difference with the intervention group showing higher TEQ total score (*p* = .008 and *p* = .005, respectively). As shown in Fig. 3, the BBB intervention group led to more overall transition preparation/success than the comparison group, both as reported by caregivers and teachers

The main effect of transition level (to elementary, middle or high school) on teacher report of transition success was marginally significant (*F*(2, 226) = 2.98, *p* = .053). Pairwise comparisons showed overall that the transition to middle school (M TEQ total score = 47.29, *SE* = 2.21) was marginally (*p* = .05) less successful than the transition to elementary school (M TEQ total score = 53.8, *SE* = 1.58), which was similar to the transition to high school (TEQ total score = 50.35; *p* = .96). The main effect of transition level on caregiver report of transition success was not statistically significant (*F*(2, 139) = 1.18, p = .31).

### Secondary Outcome: Parent Self-Efficacy and Worry in Managing the Transition to School

For caregiver self-efficacy in managing the transition to school (PSMTSS Efficacy total score), there was a intervention group main effect (*F*(1, 118) = 11.16, *p* = .001), with the intervention group having more efficacy than the comparison group across timepoints, and a time main effect (*F*(1, 191) = 43.42, *p* < .001), with both groups having more efficacy at post-intervention relate to baseline, but no intervention group effect × time interaction effect (*F*(1, 136) = 1.75, *p* = .19).

**Fig. 4:**
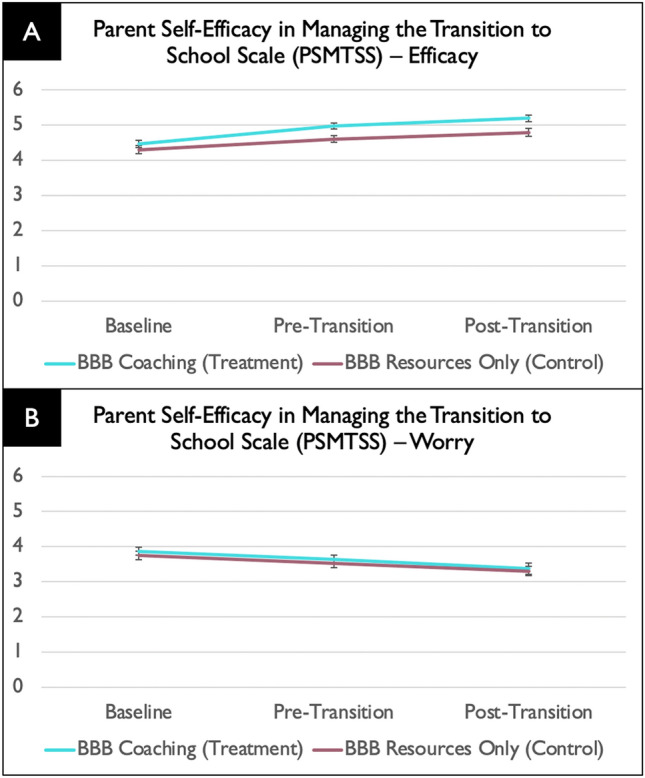
Parent Self-Efficacy in Managing the Transition to School Scale (PSMTSS) results showing higher caregiver efficacy at the Pre-Transition and Post-Transition timepoints (**A**) and a reduction in worry across time in both intervention groups (**B**)

For caregiver worry about managing the transition to school (PSMTSS Worry total score), there was a time effect (*F*(1, 225) = 25.35, *p* < .001), but no intervention group effect (*F*(1, 105) = 1.04, *p* = .31) nor intervention group effect × time interaction effect (*F*(1, 225) = 0.04, *p* = .84). As shown in Fig. 4, for both intervention groups, caregiver worry decreased throughout the transition. The main effect of student transition on caregiver’s efficacy in managing the transition to school was not significant (*F*(2, 102) = 0.84, *p* = .92).

### Implementation Outcomes

#### Coaching Fidelity

Observed-rated fidelity on the implementation of the 12 intervention fidelity items across coaches ranged from 92.20−100% (*M* = 98.71%); self (coach)-rated fidelity ranged from 96.66−100% (*M* = 99.57%).

#### Intervention Usability

**Fig. 5:**
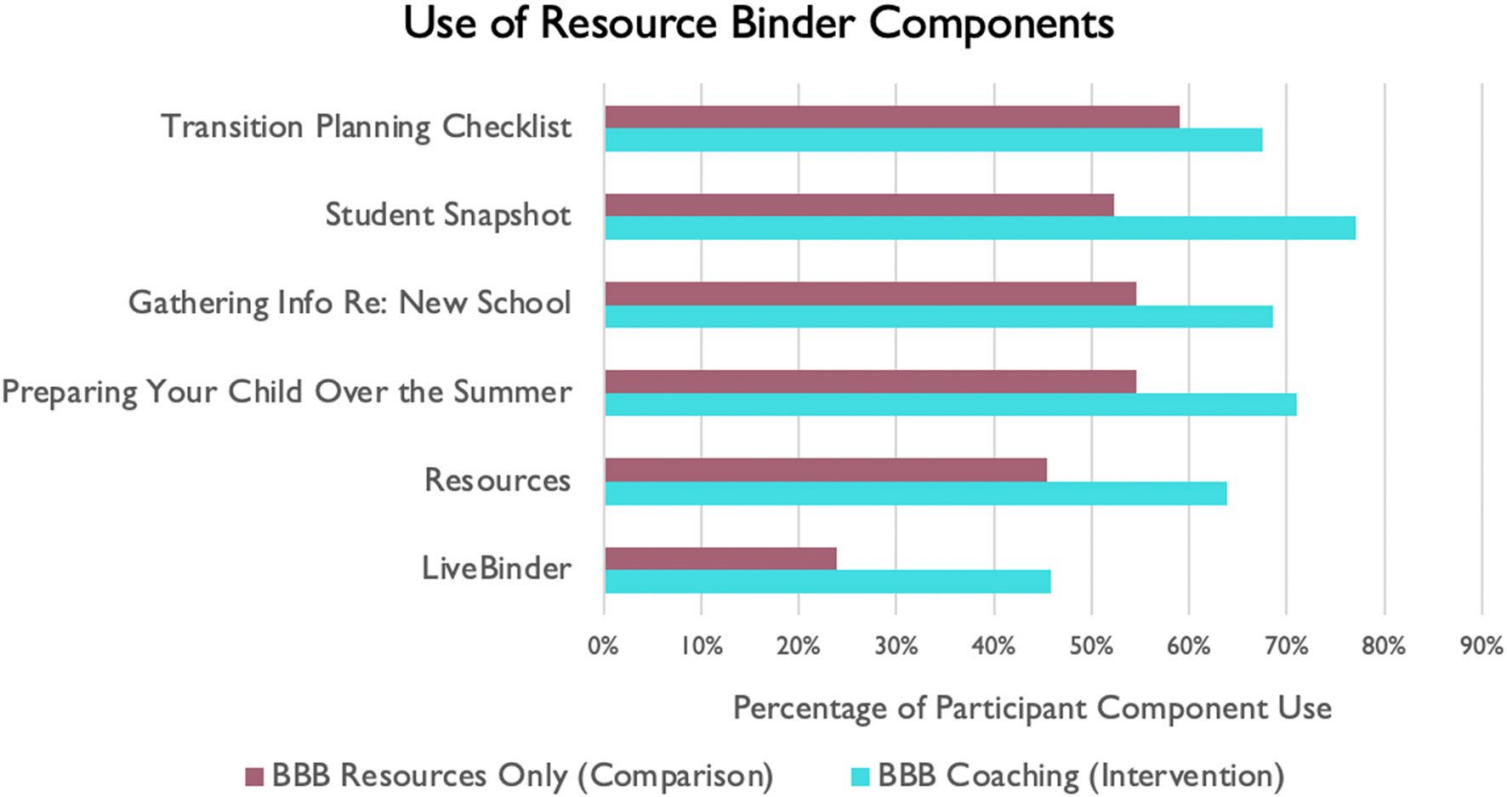
Use of resource binder components (percentage of participants) across the intervention groups

**Fig. 6:**
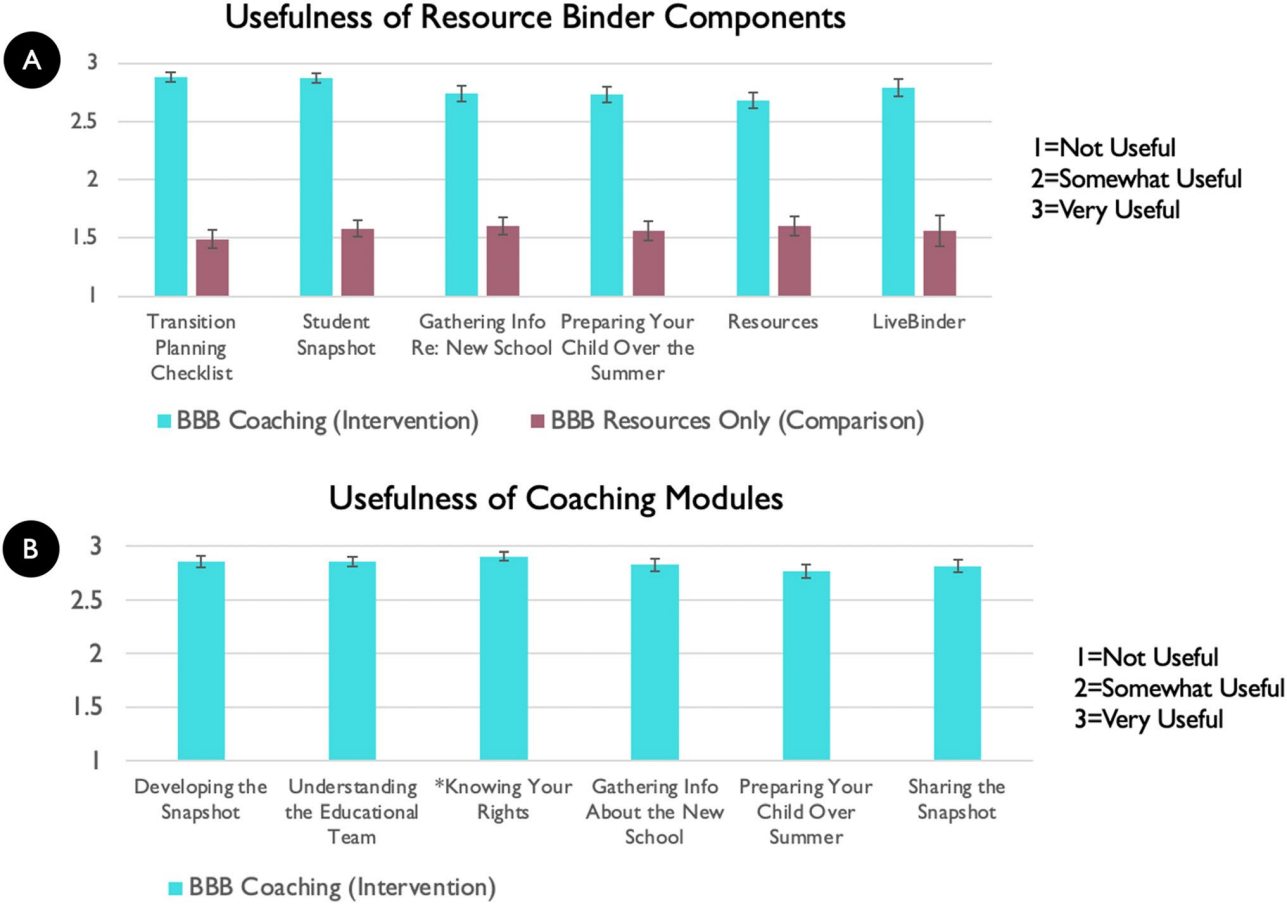
Intervention usefulness across the intervention and comparison groups of resource binder components (**A**) and coaching modules (**B**). *The Knowing Your Rights module was completed by 97.6% caregivers (was an optional module)

As shown in Figs. 5 and 6, the BBB intervention group used the BBB Resource Binder components more and also thought they were more useful than the comparison group. The intervention group also thought the coaching on the caregiver modules were very useful.

#### Intervention Acceptability

**Fig. 7:**
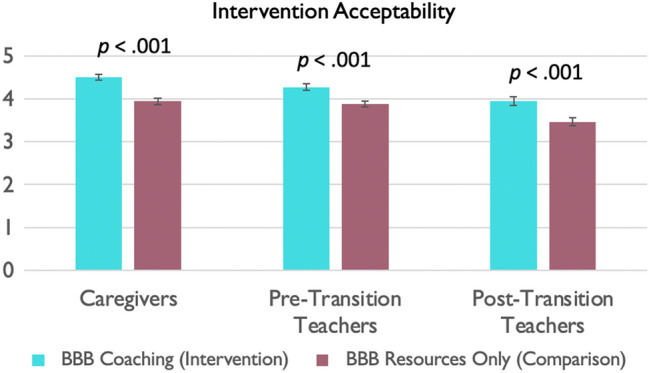
Intervention acceptability, across intervention and comparison groups

As shown Fig. 7, both groups found the interventions to be highly acceptable, though the BBB intervention group found it more acceptable than the comparison group.

#### Binder Component Usefulness Ratings and Outcomes

As shown in Supplementary Tables 1–3, higher caregiver’s perceived transition success was significantly correlated with higher caregiver-rated usefulness of the transition planning checklist and gathering information regarding the new school module in the binder, and higher teachers’ perceived transition success was marginally correlated with caregiver-rated usefulness of the transition planning checklist.

As shown in Supplementary Tables 4–6, higher caregiver self-efficacy in managing the transition to school was related with caregivers’ higher ratings of usefulness of the transition planning checklist and Livebinder, as well as pre-transition teachers’ higher ratings of usefulness of the transition planning checklist, the preparing your child over the summer binder module and Livebinder. Lower caregiver worry in managing the transition to school was related with pre-transition teachers’ (but not caregivers’) higher ratings of usefulness of the preparing your child over the summer binder module.

## Discussion

In the first randomized controlled trial of a school transition intervention, we tested the collaboratively developed BBB for students on the autism spectrum and their caregivers and teachers, within predominantly minoritized families in under-resourced communities. Findings provide empirical support for the intervention, showing that both caregivers and teachers from under-resourced school districts rated the transition post-intervention as more successful in the intervention group than in the comparison group. These findings are consistent with the only other intervention on school transitions that was tested in a quasi-experimental study (STEP-ASD; Mandy et al., 2016), showing that transition supports can make a positive impact during school transitions, and extended these findings to show that transitions interventions can be successful with minoritized families in under-resourced communities and throughout the transition to elementary school.

This study highlights the vital importance of empowering caregivers, specifically of under-resourced families, by giving them tools and information to advocate and participate in this important transitional time. Our caregiver and teacher report findings are consistent with past research showing caregiver’s involvement as the active ingredient in their child’s intervention gains (Bearss et al., 2015; Kasari et al., 2014; Wetherby & Woods, 2006).

We used the RE-AIM framework as a guiding framework for the study. We targeted *Reach* by examining our ability to hit our recruitment targets among minoritized families, including families of color and families with limited resources. Future studies should examine and further improve *Reach* beyond study participation. We examined the *Efficacy* of the intervention in this study and *Adoption and Implementation* through the measures of usability and acceptability in this study. We found that most participants used the intervention materials and found them and the coaching very useful. Caregivers in both groups, but especially the intervention group, found the intervention to be highly acceptable. These results are encouraging and suggest that the BBB intervention is a good fit for families living in low-income households transitioning to public schools and supports wider dissemination efforts and further studying of *Adoption and Implementation*, and *Maintenance* of the BBB program. Sustaining this intervention at scale will require determining who would take on the caregiver Transition Coach role. For this study, the person in that role was a part of the research team but would need to be embedded into the school district or paid for within school district budgets in order for the intervention to maintain long term. This may be especially difficult to navigate in the preschool to elementary school transition as many students on the autism spectrum transition from early intervention preschools that operate outside of the school district. One potential solution would be to assign the Transition Coach as soon as the child is signed up for kindergarten which for many school districts is well in advance of the transition. Future research is needed to determine the best implementation strategies to support districts in implementing the BBB program with their own resources.

The optimal delivery format also should be explored; given the context of the recent global COVID-19 pandemic, an online format may be preferable to teachers and caregivers instead of an in-person format. We found that flexibly offering both options allowed for the best engagement with teachers and caregivers. There may also be a better way to deliver binder content, for example, use of an app that caregivers and teachers could use on their phone rather than a Livebinder which is optimized for computer use, to further bolster the binder’s impact.

Although caregivers in both the BBB and comparison groups experienced some worry before the transition, both groups’ worry gradually decreased throughout the course of the school transition. These findings contrast with recent reviews (Fontil et al., 2020; Nuske et al., 2019) that show high caregiver worry during school transitions for students on the autism spectrum, which may suggest that the transition resource binder provided to both intervention and comparison groups provided some comfort during this sensitive period of change. However, given there was only moderate binder-use among participants in the intervention group (and moderate to low in the comparison group), this decrease may be more to do with external factors such as the simple passage of time. Nevertheless, the potential for an additional caregiver module focusing on decreasing caregiver worry/stress should be considered in future research.

Our analyses on the relationship between binder component usefulness ratings and outcomes further highlighted the importance of engaging with transition planning checklist in particular for bolstering transition success, caregiver self-efficacy in managing the new school transition, and limiting caregiver worry throughout the process. Engagement with the Livebinder was highlighted also as helpful for supporting caregiver self-efficacy and minimizing caregiver worry. These binder components were introduced to help participants get organized around the transition process and allow for easy access of binder material. Further efforts to emphasize these components, for example, as suggested above, to introduce an intervention app to house all binder components, may be useful. Further research is needed to explore this as an implementation strategy to increase engagement with the intervention.

There was a discrepancy between caregivers in the intervention group reporting, one the one hand, more transition success (relative to success reported by caregivers in the comparison group), and on the other hand, no change in their own self-efficacy in managing the transition to a new school (relative to the change caregivers experienced in the comparison group). This finding may perhaps reflect them not feeling confident in their newfound advocacy skills throughout the transition process despite success in doing so. These findings may indicate that further coaching techniques or perhaps adding a coaching module specific to self-efficacy in parental advocacy may be needed to help to bolster caregivers’ confidence in managing their child’s school transitions. Future research should address how to further support self-efficacy in caregivers in managing their children’s new school transitions.

Given that use of the Transition Planning Checklist was relatively and similarly high in the intervention and comparison groups, this indicates that less coaching is needed for caregiver with this intervention component and could be considered as a standalone, cheap intervention for schools to uptake (e.g., giving to all caregivers of students transitioning to the new school early in the pre-transition year). Future research is needed to explore this further.

Interestingly, teachers marginally reported lower transition success to middle school (relative to kindergarten and high school), whereas parents did not note any differences in transition success by transition level. Middle school can represent a difficult period of adjustment for children as they navigate the transition from childhood into early adolescence, which may be particularly pronounced in children on the autism spectrum are at higher risk of mental health struggles (Rodriguez et al., 2021). This may explain the teacher finding, and may indicate that parents are not seeing children’s struggles at home as are teachers are at school. Further research is needed to elucidate this issue.

The extent to which the results reported herein generalize to other populations and settings is unknown. Future research should aim to address, for example, if this intervention would be helpful with children who have intellectual disability without autism or in schools outside of the US. Further, in order to examine implementation strategies to bolster caregiver and teacher engagement, and to examine the long-term impact, sustainability and scale up of the intervention a hybrid efficacy-implementation trial as a next step is warranted.

## Limitations

Several study limitations should be considered. First, the participants were not naive to the intervention group; therefore, respondents may have answered questions based on their knowledge of receiving the intervention (i.e., coaching) versus the caregiver module and resource binder only. We did not use more objective measures or direct child measures of transition success as to our knowledge no such measures exist. The concept of transition success has not been well-defined in the literature, with some scholars focusing on wide-ranging aspects that touch transition success like child behavior to teachers’ knowledge of new students. More conceptual work is needed to ascertain conceptual boundaries around transition success and to determine if the measure we used to capture successful transition planning and transitions map onto more objective measures. Once established, such objective and direct measures would be helpful in reducing potential bias by parents and teachers in filling out transition success outcome measures as they naturally could not be blind to intervention group allocation. Second, the primary and secondary outcome measures relied on caregiver and teacher reports and did not use systematic observations of the students in pre- or post-transition settings. Third, the primary outcome was self-developed so it was not a validated measure, though reliability statistics were promising. Fourth, it should be noted that pre- and post-transition teachers were different, which introduces measurement error (but is inherent in the issue of school transition studies). Pre-transition teachers have limited time and resources and are unable to follow past students into their new school, therefore they were not able to answer post-transition outcome measures. Fifth, it should also be noted that we had a moderate attrition rate for caregivers (20.6%). For some caregivers, practical or eligibility reasons deemed them unable to participate (family moved out of state, decided to keep their child in preschool for an additional year), for others, they were not able to maintain regular coaching and check-ins calls. Due to the mix of different reasons for attrition is a difficult to ascertain how this may have impacted results. Regardless, future research should examine strategies to minimize attrition, such as additional financial compensation or further procedures to match coaches to caregivers. Sixth, regarding coaching fidelity although we included interrater reliability measures for 20% of sessions, these fidelity checks were based on self-report which does introduce potential for bias. Future work should examine strategies to keep caregivers engaged while minimizing the time commitment. Seventh, there was potential cross-contamination across classes since parents in different classes may have talked to each other. Finally, our intervention arms evaluated the additive effect of having coaching, but we did not elucidate in our study *how* coaching was helpful (i.e., was it coaching and feedback, social support, increased exposure to the binder content, facilitating collaboration with teachers, or something else). Further research could elucidate this issue by asking parents specifically how the coaching impacted transition success.

## Conclusion

Results suggest that the BBB caregiver coaching intervention, as opposed to access to the caregiver modules and resource binder without the coaching component, can improve overall transition planning and success for under-resourced families and racially/ethnically diverse students on the autism spectrum. Results also suggest that the intervention is generally usable and acceptable to teachers and caregivers. Findings support potential dissemination to school personnel, however as this is the first randomized controlled trial on the intervention, findings warrant replication, and strategies to support adoption, implementation and maintenance in schools are needed.

## Supplementary Information

Below is the link to the electronic supplementary material.

Supplementary material 1 (DOCX 26.9 kb)
