# Supplementary material for: Building Better Bridges: Outcomes of a Community-Partnered New School Transition Intervention for Students on the Autism Spectrum
Source: J Autism Dev Disord. 2024 Jun 12;55(9):3100–17. doi: 10.1007/s10803-024-06285-7 (PMC11972019; doi:10.1007/s10803-024-06285-7)
Supplement: Supplementary file 1 — Supplementary material 1 (DOCX 26.9 kb) [file 10803_2024_6285_MOESM1_ESM.docx]

**Supplementary Material**

Intervention Acceptability Scale………………………………………………………………………….... page 2

Pearson Correlations between Perceived Transition Success and:

**Supplementary Table 1.** *Caregivers’* *Binder Usefulness Ratings……………………………………..*page 3

**Supplementary Table 2.** *Pre-Transition Teachers’ Binder Usefulness Ratings…………………..…*page 3

**Supplementary Table 3.** *Post-Transition Teachers’ Binder Usefulness Ratings…………………….*page 3

Pearson Correlations between Caregiver Efficacy/Worry in Managing the School Transition and:

**Supplementary Table 4.** *Caregivers’* *Binder Usefulness Ratings………………………………………*page 4

**Supplementary Table 5.** *Pre-Transition Teachers’ Binder Usefulness Ratings*……………………page 4

**Supplementary Table 6.** *Post-Transition Teachers’ Binder Usefulness Ratings*…………………..page 4

**Intervention Acceptability Scale**

(1) I feel that the intervention was an appropriate intervention for this child;

(2) I feel that the intervention improved this child’s transition;

(3) I feel that the intervention was easy to implement;

(4) I would recommend this intervention to other parents;

(5) I am satisfied with this intervention;

(6) I would use the tools from this intervention with this child or other children in the future;

(7) I found it easy to access the intervention materials;

(8) Overall, I found the Building Better Bridges intervention to be an easy intervention to do;

(9) Overall, I found the Building Better Bridges intervention helpful for my child's transition;

(10) Overall, I believe the Building Better Bridges intervention was effective for my child.

Items were scored on a 5-point Likert scale: 1= Strongly disagree, 2 = Disagree, 3 = Don’t Know/Neutral, 4 = Agree, 5 = Strongly Agree, such that where higher scores indicate higher acceptance of the intervention.

Adapted from the intervention acceptability sub-scale used in Shih et al. (2019):

Shih, W., Dean, M., Kretzmann, M., Locke, J., Senturk, D., Mandell, D. S., Smith, T., & Kasari, C. (2019). Remaking recess intervention for improving peer interactions at school for children with autism spectrum disorder: Multisite randomized trial. *School Psychology Review*, *48*(2), 133–144.

**Supplementary Table 1**

*Pearson Correlations between Perceived Transition Success* *and* ***Caregivers’*** *Binder Usefulness Ratings*

|  | **Usefulness Ratings** | | | | | |
| --- | --- | --- | --- | --- | --- | --- |
|  | **Transition Planning Checklist** | Student Snapshot | **Gathering Info Re New School** | Preparing Your Child Over the Summer | Resources | LiveBinder |
| **Caregiver TEQ** | **.20^*^** | 0.09 | **.20^*^** | 0.09 | 0.16 | 0.15 |
| **Teacher TEQ** | **0.19^#^** | 0.13 | 0.01 | 0.10 | 0.09 | -0.01 |

*Note.* TEQ = Transition Evaluation Questionnaire. Positive correlations represent higher usefulness ratings = better transition success. *^**^p < .*001*, ^*^p < .*05*, ^#^p < .*10.

**Supplementary Table 2**

*Pearson Correlations between Perceived Transition Success and* ***Pre-Transition Teachers’*** *Binder Usefulness Ratings*

|  | **Usefulness Ratings** | | | | | |
| --- | --- | --- | --- | --- | --- | --- |
|  | Transition Planning Checklist | Student Snapshot | Gathering Info Re New School | Preparing Your Child Over the Summer | Resources | LiveBinder |
| **Caregiver TEQ** | 0.08 | -0.16 | 0.07 | 0.20 | 0.23 | -0.05 |
| **Teacher TEQ** | 0.11 | 0.05 | -0.12 | -0.03 | 0.07 | 0.11 |

*Note.* TEQ = Transition Evaluation Questionnaire. Positive correlations represent higher usefulness ratings = better transition success. *^**^p < .*001*, ^*^p < .*05*, ^#^p < .*10.

**Supplementary Table 3**

*Pearson Correlations between Perceived Transition Success and* ***Post-Transition Teachers’*** *Binder Usefulness Ratings*

|  | **Usefulness Ratings** | | | | | |
| --- | --- | --- | --- | --- | --- | --- |
|  | Transition Planning Checklist | Student Snapshot | Gathering Info Re New School | Preparing Your Child Over the Summer | Resources | LiveBinder |
| **Caregiver TEQ** | -0.228 | -0.087 | -0.144 | -0.003 | 0.190 | 0.090 |
| **Teacher TEQ** | 0.054 | 0.146 | -0.011 | -0.010 | 0.065 | -0.222 |

*Note.* TEQ = Transition Evaluation Questionnaire. Positive correlations represent higher usefulness ratings = better transition success. *^**^p < .*001*, ^*^p < .*05*, ^#^p < .*10.

**Supplementary Table 4**

*Pearson Correlations between Caregiver Self-Efficacy/Worry in Managing the School Transition and* ***Caregivers’*** *Binder Usefulness Ratings*

|  | **Usefulness Ratings** | | | | | |
| --- | --- | --- | --- | --- | --- | --- |
|  | **Transition Planning Checklist** | Student Snapshot | Gathering Info Re New School | Preparing Your Child Over the Summer | Resources | **LiveBinder** |
| **PSMTSS - Efficacy** | **0.170^#^** | 0.107 | 0.069 | -0.021 | 0.092 | **.343^**^** |
| **PSMTSS**  **- Worry** | -0.012 | 0.069 | -0.141 | -0.030 | -0.034 | -0.021 |

*Note.* PSMTSS = Parental Self-efficacy in Managing the Transition to School Scale. Positive correlations represent higher usefulness ratings = higher self-efficacy/worry. *^**^p < .*001*, ^*^p < .*05*, ^#^p < .*10.

**Supplementary Table 5**

*Pearson Correlations between Caregiver Self-Efficacy/Worry in Managing the School Transition and* ***Pre-Transition Teachers’*** *Binder Usefulness Ratings*

|  | **Usefulness Ratings** | | | | | |
| --- | --- | --- | --- | --- | --- | --- |
|  | **Transition Planning Checklist** | Student Snapshot | Gathering Info Re New School | **Preparing Your Child Over the Summer** | **Resources** | **LiveBinder** |
| **PSMTSS - Efficacy** | **0.214^#^** | 0.007 | 0.079 | **.332^*^** | **.372^**^** | **0.229^#^** |
| **PSMTSS**  **- Worry** | -0.044 | -0.069 | -0.134 | **-.309^*^** | -.321^*^ | -0.037 |

*Note.* PSMTSS = Parental Self-efficacy in Managing the Transition to School Scale. Positive correlations represent higher usefulness ratings = higher self-efficacy/worry. *^**^p < .*001*, ^*^p < .*05*, ^#^p < .*10.

**Supplementary Table 6**

*Pearson Correlations between Caregiver Self-Efficacy/Worry in Managing the School Transition and* ***Post-Transition Teachers’*** *Binder Usefulness Ratings*

|  | **Usefulness Ratings** | | | | | |
| --- | --- | --- | --- | --- | --- | --- |
|  | Transition Planning Checklist | Student Snapshot | Gathering Info Re New School | Preparing Your Child Over the Summer | Resources | LiveBinder |
| **PSMTSS - Efficacy** | -0.079 | -0.067 | 0.046 | -0.190 | -0.026 | 0.004 |
| **PSMTSS**  **- Worry** | 0.145 | -0.154 | 0.230 | 0.058 | -0.036 | -0.248 |

*Note.* PSMTSS = Parental Self-efficacy in Managing the Transition to School Scale. Positive correlations represent higher usefulness ratings = higher self-efficacy/worry. *^**^p < .*001*, ^*^p < .*05*, ^#^p < .*10.
